# Adapting TeamSTEPPS for school mental health teams: development of an implementation research logic model

**DOI:** 10.3389/frhs.2025.1675020

**Published:** 2025-12-18

**Authors:** Jordan Albright, Suzanne S. Tham, Biiftu Duresso, Samantha Rushworth, Aparajita Biswas Kuriyan, Ricardo B. Eiraldi, Courtney Benjamin Wolk

**Affiliations:** 1 Department of Psychology, University of South Alabama, Mobile, AL, United States; 2 Center for Mental Health, University of Pennsylvania Perelman School of Medicine, Philadelphia, PA, United States; 3 School of Social Policy & Practice, University of Pennsylvania, Philadelphia, PA, United States; 4 Department of Pediatrics and Psychiatry, University of Pennsylvania Perelman School of Medicine, Philadelphia, PA, United States; 5 Department of Child and Adolescent Psychiatry and Behavioral Sciences, Children’s Hospital of Philadelphia, Philadelphia, PA, United States; 6 Leonard Davis Institute of Health Economics, University of Pennsylvania, Philadelphia, PA, United States

**Keywords:** school mental health, implementation research logic model, determinants, implementation strategies, team science

## Abstract

**Introduction:**

Multidisciplinary school mental health (SMH) teams play a key role in delivering mental health services to children. However, poor workflow, inefficient communication, and limited resources, compromise SMH service delivery. Despite robust literature demonstrating the efficacy of team science interventions, such as the Team Strategies and Tools to Enhance Performance and Patient Safety (TeamSTEPPS), research on these interventions with SMH teams is limited.

**Methods:**

We conducted qualitative interviews with SMH team members, teachers, and school administrators who had participated in a hybrid effectiveness-implementation trial of TeamSTEPPS. Participants identified barriers and facilitators to implementation of the adapted TeamSTEPPS intervention, which were then organized according to the Consolidated Framework for Implementation Research (CFIR). An Implementation Research Logic Model was developed, aligning implementation determinants with implementation strategies and proposed mechanisms by which the strategies impact outcomes.

**Results:**

Barriers to the successful implementation of the adapted TeamSTEPPS intervention included a lack of financing and resources, the intervention not being a relative priority, mission misalignment, poor work infrastructure to support, unmotivated innovation recipients and leaders, and insufficient planning. Proposed implementation strategies included providing dynamic training for leadership and SMH team members, centralizing technical assistance, development and distribution of educational materials, and ongoing consultation about implementation supports/when challenges arose, developing local policies that support implementation, establishing mandates for change, pruning competing initiatives, and providing reminders of strategies to school personnel. Proposed implementation outcomes (e.g., acceptability, feasibility), service outcomes (e.g., Observation of use of TeamSTEPPS strategies, Perceptions of teaming, Attitudes toward teamwork), and “client outcomes” (e.g., student service use, absences, suspensions, grade promotion) were also identified.

**Discussion:**

Lessons from the implementation process and recommendations for future directions are highlighted to inform the delivery and sustainment of team science interventions, such as TeamSTEPPS, for use with SMH teams.

## 1 Introduction

Schools are the primary access point for mental health services for children in the United States (1, 2). School mental health (SMH)^1^ teams play a key role in delivering prevention, early intervention, and treatment services, often through multi-disciplinary teams of professionals (e.g., social workers, school counselors, school psychologists) who collaborate closely with other school personnel (e.g., teachers, school leadership) and community organizations to meet students' mental health needs (3). However, due to often fluid structures, SMH teams are vulnerable to poorly defined workflows, inefficient or limited communication, undefined roles, insufficient time for collaboration, and limited resources (4–7). These challenges, if not addressed, can lead to disorganized and ineffective service provision.

While the literature on team science and multidisciplinary teaming demonstrates the importance of effective collaboration for efficient, productive, high-quality team service delivery (8–10), effective and efficient multidisciplinary collaboration remains challenging for SMH teams (5). Research on team-based care has primarily focused on the characteristics of effective teams and their impact on service outcomes (11, 12). This literature has not been translated into feasible, acceptable, and effective team science implementable interventions (13), and the literature on contextual factors impacting school-based team care delivery is limited.

There is a robust literature demonstrating the efficacy of team-based interventions outside of SMH (14–19]), and a limited but growing body of literature demonstrating the potential for team science interventions to improve SMH team dynamics (14, 20–23). Team Strategies and Tools to Enhance Performance and Patient Safety [TeamSTEPPS (24)] is a widely used and studied team training intervention, originally developed and disseminated in healthcare settings (25–27). TeamSTEPPS focuses on building teamwork competencies in leadership, situation monitoring, communication, and mutual support through a training curriculum and a suite of tools that integrate these principles into the service setting.

Studies have found that participation in TeamSTEPPS improves team function *and* patient outcomes in healthcare settings (15, 26, 28, 29). While adaptations of TeamSTEPPS in non-healthcare environments are limited, recent pilots of adapted TeamSTEPPS models are demonstrating its potential in novel service settings (13, 21, 22), including with SMH teams (14). These efforts are supported by findings that suggest TeamSTEPPS' core effectiveness is highly reliable across different trainee groups and methods, confirming that its underlying principles can be successfully applied to diverse team-based settings (15). The current study leverages an ongoing hybrid effectiveness-implementation trial of an adapted TeamSTEPPS curriculum and implementation supports with SMH teams to explore contextual factors that may impact implementation of teamwork interventions. This is important as few studies have examined perspectives of SMH team members, teachers, and administrators regarding challenges in implementing team-science interventions.

This summative assessment addresses this gap in the literature by presenting findings from qualitative interviews with participants in an adapted TeamSTEPPS intervention. Barriers and facilitators are organized according to the Consolidated Framework for Implementation Research [CFIR (30)] to provide a comprehensive understanding of implementation determinants. While the CFIR has been widely used to identify factors impacting the implementation of evidence-based practices (EBPs) in schools (31–34), previous studies have largely targeted the student population. This is the first study, to our knowledge, using CFIR to identify factors impacting the implementation of a team science intervention for SMH teams. We also provide the final version of an iteratively refined Implementation Research Logic Model (IRLM), which aligns these implementation determinants with coinciding implementation strategies. The IRLM also identifies proposed mechanisms by which the strategies impact implementation-, system-, and student-level outcomes. Specifically, the mechanisms proposed in the IRLM are grounded in team science literature, which suggests that team training, like the adapted TeamSTEPPS, is effective through a sequential pathway in which improved team member learning leads to skill transfer in the work environment, which ultimately results in positive service outcomes (15). IRLMs comprehensively examine factors impacting the implementation and sustainment of EBPs, allowing for a standardized approach to examining these processes over time. The goal of the present study is to illustrate implementation determinants in accordance with CFIR and contextualize these factors within an IRLM to inform future implementation and sustainment of team science interventions with SMH teams.

## 2 Materials and methods

The research was reviewed and approved by the [BLINDED] Institutional Review Board. Informed consent was obtained prior to engagement in research activities. Participants were informed that interview audio files would only be accessible to members of the research team. Once interviews were transcribed, audio recording files would be disposed of, and interview transcriptions would be de-identified to maintain confidentiality. We report qualitative methods and findings according to the consolidated criteria for reporting qualitative studies [COREQ (35)]. Additional details about the larger trial are available in Kuriyan et al. (36).

### 2.1 Participants

SMH team staff (e.g., clinical team leadership, clinicians, paraprofessionals), teachers, and school administrators (e.g., principals, special education coordinators) from eight public and charter schools in one Mid-Atlantic metropolitan area who had participated in a hybrid effectiveness-implementation trial of TeamSTEPPS were recruited to participate in qualitative interviews. Class size varied by school.

### 2.2 Measures

Demographic information was obtained using a standardized record form.

#### 2.2.1 Interview Guide

A semi-structured interview guide (see Supplementary Appendix A) was developed, containing questions about the feasibility and acceptability of TeamSTEPPS and contextual influences (e.g., leadership engagement, alignment with existing team culture). Additionally, a final set of questions asked participants to reflect on their responses on previously administered questionnaires about inter-professional collaboration, team skills, and behavior. The interview guide underwent iterative refinement with research team members to ensure its applicability and suitability before deployment.

### 2.3 Procedure

Simple random sampling procedures were used, such that participants from the larger trial subject pool were randomly contacted via email and invited to complete a semi-structured qualitative interview. Thirty participants were contacted, 15 did not respond, 14 completed an interview, and one participant initially agreed but became unresponsive. Participants were compensated $50 for their participation.

A bachelor's-level research assistant and postdoctoral research fellow, both with training in qualitative interviewing and data collection, conducted individual interviews via Zoom between May and July 2023. Interviews lasted 47 min on average (00 : 29:33–01:01:55).

### 2.4 Data analysis

All interviews were audio-recorded, transcribed, and uploaded into NVivo 14 software for data management. We utilized an integrated analytic approach (37). Through an iterative process, the research team developed a qualitative codebook, which included predetermined attributes of interest from the CFIR (30), components of the TeamSTEPPS intervention, and emergent codes and themes (e.g., barriers, facilitators; see Table 1 for description of codes). First, two research team members independently coded two initial transcripts. The coding team then met to discuss points of agreement, address discrepancies through consensus discussion, and refine the codebook. The two research team members then reviewed an additional two transcripts based on the refined codebook and met again with the research team for consensus discussion regarding coding discrepancies. At this stage, the interrater reliability yielded a Kappa score ≥0.9, indicating excellent interrater agreement. The two research team members then independently coded.

**Table 1:** Characteristics of qualitative interview participants.

Characteristic	Interview *n (%)*
Total Participants	14
Gender	
Female	13 (92.9)
Prefer to Self-describe	1 (7.1)
Race	
White	11 (78.6)
Black	2 (14.3)
Prefer not to disclose	1 (7.1)
Ethnicity	
Non-Hispanic/Latinx	13 (92.9)
Hispanic/Latinx	1 (7.1)
Participant Roles	
Guidance Counselor	4 (28.6)
School Leadership	3 (21.4)
School Psychologist	3 (21.4)
Social Worker	3 (21.4)
Teachers	1 (7.1)
Participant Affiliation	
Partner 1	11 (78.6)
Partner 2	2 (14.3)
Partner 3	1 (7.1)
Licensure Status	
Licensed	7 (50.0)
In-Progress	2 (14.3)
No	5 (35.7)
Provided Clinical Services	2 (14.2)

Participants on average were 41.57 years old (*SD* = 9.8). On average, participants worked in their current school for 9.0 years (*SD* = 9.6), the district for 10.8 years (*SD* = 9.4), and their respective fields for 14.6 years (*SD* = 9.8).

### 2.5 Implementation research logic model

An *a priori* Implementation Research Logic Model [IRLM (38)] was developed to guide the hybrid effectiveness-implementation trial of TeamSTEPPS. This model was created with input from an advisory board, which helped shape implementation procedures, as well as the research team, which included investigators, TeamSTEPPS trainers, and qualitative coding team members. The IRLM was iteratively refined over the course of the research project via consensus discussion amongst members of the research team. The final iteration of the IRLM was further informed by key insights from participants regarding implementation determinants.

Barriers and facilitators identified by participants were contextualized within the CFIR and depicted in the IRLM as “Determinants.” These determinants were ordered sequentially from outer setting determinants to implementation process related factors. To inform future implementation efforts, “Implementation Strategies” were selected from the Expert Recommendations for Implementing Change (ERIC) School Implementation Strategies, Translating ERIC Resources [SISTER (39)]. Strategies were organized into phases aligned with the Exploration, Preparation, Implementation, and Sustainment (EPIS) framework (40). Implementation, service, and client-level outcomes were then identified and organized according to the Implementation Outcomes Framework [IOF (41)].

## 3 Results

Participants were primarily non-Hispanic/Latinx (92.9%), White (78.6%) females (92.9%) from a variety of professional disciplines, including school counselors (28.6%; *n* = 4), administrators (21.4%; *n* = 3), school psychologists (21.4%; *n* = 3), social workers (21.4%; *n* = 3), and a teacher (7.1%). Additional participant characteristics can be found in Table 2.

**Table 2:** Code descriptions.

Code	Definition
Outer Setting	Economic, political, and social context surrounding the school (i.e., school district, context surrounding the school, pandemic, government funding, and the socio-political landscape).
	A broad construct that includes external strategies to spread interventions, including policy and incentives, and the population served by the school.
Inner Setting	Encompasses structural, political, and cultural characteristics within schools.
	Factors include school size, staff collaboration and communication, relationship among staff members, workflow, readiness for implementation (e.g., leadership engagement, resource availability, access to information and knowledge), and implementation climate (e.g., perceived need for change, relative priority and importance within the organization and acknowledgment of other tasks that may take priority, compatibility of the intervention with existing workflows and systems), and current practice protocols and procedures regarding student services.
Individual Characteristics	
Administration	A broad construct concerning the individual characteristics of administrative team member, including district or school administration/leadership, principals, and assistant principals.
	Includes a variety of characteristics of the school or district administration that constitute determinants of implementation. These characteristics include administrator support, knowledge, attitudes, beliefs, preferences, and motivation.
SMH Team Members	A broad construct concerning the individual characteristics of any member of the school services team (also known as the student support team, child study team, pupil services team, or mental health clinicians, teachers, or other staff involved in student services).
	Includes a variety of provider characteristics that constitute determinants of implementation. These attributes include staff knowledge, attitudes, beliefs, comfort levels, preferences regarding team function, self-efficacy, and motivation.
The Innovation	Perceptions of aspects related to TeamSTEPPS content and strategies.
	These include perceptions of strength and quality of evidence supporting TeamSTEPPS, perceptions of the relative advantage of implementing TeamSTEPPS strategies, adaptability of individual TeamSTEPPS strategies, complexity of implementing TeamSTEPPS, duration of training sessions, and cost.
Implementation Process	Perceptions of the Implementation Process and methods used to implement and sustain TeamSTEPPS strategies within the school.
	Active support process aimed to facilitate the intended use of the strategies at both the individual and organizational levels.
	These include multiple subprocesses, including planning, goal setting, teaming (i.e., consulting with academic partners, joining together, intentionally coordinating, and collaborating on interdependent tasks), engaging individuals (e.g., champions or advocates of TeamSTEPPS), executing, and reflecting/evaluating.
Barrier	Cited barriers to effective teamwork, communication, and implementation of TeamSTEPPS in schools. In addition to barriers to scheduling, coordination, and execution of the strategies, this code includes more general barriers to effective communication, teamwork, and implementation of TeamSTEPPS. Barriers should be inherent to the context, such as the team or school environment.
	This code includes hypothetical or suggested barriers. Codes should demonstrate actionable barriers that impeded effective teamwork, communication, or implementation of TeamSTEPPS.
Facilitator	Cited facilitators to effective teamwork, communication, and implementation of TeamSTEPPS in schools This may include anything that participants state makes it easier to implement their chosen strategies including a supportive principal, a champion of the intervention, time to plan, effective teamwork, etc. Facilitators should be inherent to the context, such as the team or school environment, not including any involvement of the academic partners.
	This code includes hypothetical or suggested facilitators. Codes should demonstrate actionable facilitators that contributed to effective teamwork, communication, or implementation of TeamSTEPPS

### 3.1 Implementation determinant identification

**Figure 1:**
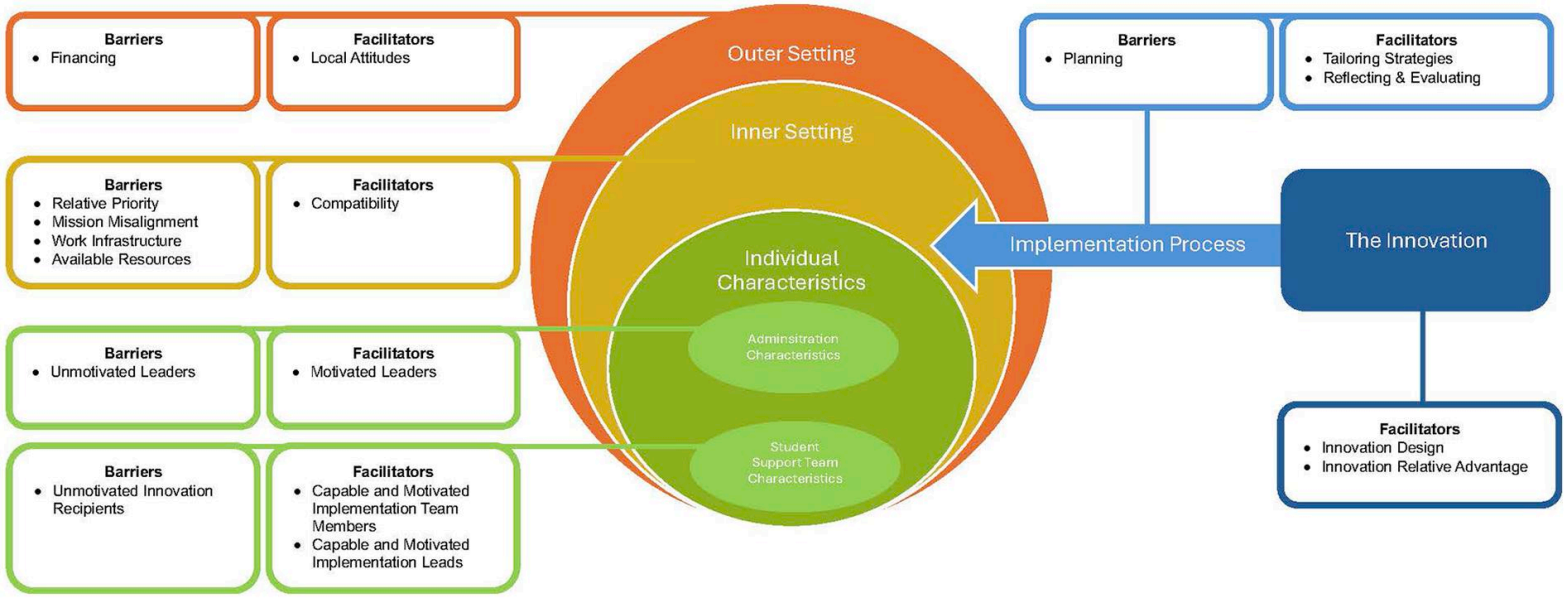
Barriers and facilitators organized according to consolidated framework for implementation research (CFIR) domains.

Barriers and Facilitators to the implementation of a team training intervention for school mental health teams, TeamSTEPPS, organized according to CFIR domains, follow. For additional details, see Figure 1.

#### 3.1.1 Outer setting

##### 3.1.1.1 Barriers

###### 3.1.1.1.1 Financing

While not directly related to TeamSTEPPS implementation, participants noted that limited school district resources and funding caused staff to feel overwhelmed, which they perceived as a barrier to implementing new initiatives.

##### 3.1.1.2 Facilitators

###### 3.1.1.2.1 Local attitudes

Participants recognized the importance of the intervention and support for training in TeamSTEPPS by district leadership, which helped to facilitate implementation. One participant shared that district leadership's participation conveyed to staff that teamwork topics are a priority. This invited team members to engage in efforts to improve team functioning.

The Principal illustrated this point, sharing, “*[Team Member] was a brand-new school psychologist this year. So, he came in, you know not knowing our systems and processes. So, of course, coming in and seeing what we’re doing is like, ‘Oh my gosh,’ there's definitely, you can see, room for improvement. And then you have TeamSTEPPS that comes in and just kind of reaffirms that, and it's like okay, now this is the district giving us this training. They want us to utilize this. Now we can move forward.”*

#### 3.1.2 Inner setting

##### 3.1.2.1 Barriers

###### 3.1.2.1.1 Relative priority

Due to competing demands in the school environment, participants found it challenging at times to prioritize and implement TeamSTEPPS strategies. As the Teacher shared, “*Because I think everybody wants a school to be successful, right? Like, a school is a community space. So, everyone wants that community space to be successful, which is the ultimate goal, but the lanes to get there for everyone just looks very different all the time. And I think … those competing priorities often are the things that cause conflict.”*

Relatedly, Guidance Counselor 3indicated that balancing the demands of school initiatives can be challenging, sharing, “*I think especially in our school district and probably other similar school districts [there] are like a lot of initiatives, and they usually don*’*t succeed because of the lack of follow through.”*

###### 3.1.2.1.2 Mission misalignment

Several participants identified a misalignment between the intervention and their administration's overarching commitment. School administrators (e.g., principals, vice principals, special education coordinators) often determine how roles and responsibilities are distributed. As such, a lack of awareness and commitment by some administrators made implementation challenging.

The Team Leader illustrated this point, sharing, “*It's just that upper administration and even, like, principal level, building level administrators are, you know, it's tough. I mean, it's tough to implement something when the leader in your building or your group or like the people who are supervising you don*'t know what you're talking about or why you're trying to implement it or understand that. people that make decisions and need to be invested for anyone else to be invested.”

###### 3.1.2.1.3 Work infrastructure

Some TeamSTEPPS strategies were intended to be used in interdisciplinary team settings, which include team members from various levels of administration. Participants shared that navigating the administrative hierarchy within schools can be hard; as such, some of the TeamSTEPPS strategies lost their effectiveness. Guidance Counselor 3 illustrated this point when sharing, “*Not everyone feels empowered to go to higher ups to even ask a question.”*

###### 3.1.2.1.4 Available resources

Participants also indicated that a lack of available resources in the form of consistent personnel from year to year (i.e., high staff and administrator turnover) could negatively impact implementation as well as future sustainment efforts. When involved personnel leave, the institutional knowledge of the intervention and the coinciding strategies leave with them.

The Teacher shared how administrative turnover challenged implementation, stating, “*There was some turnover in leadership, and I think that when there's turnover in leadership and then there isn't someone that's immediately capable of doing a job afterwards, that was just up in the air, which made everyone*'s roles throughout the course of the year a lot difficult.”

##### 3.1.2.2 Facilitators

###### 3.1.2.2.1 Compatibility

Several characteristics of the existing school service infrastructure facilitated the implementation of TeamSTEPPS strategies. For example, having identified teams or groups to focus the TeamSTEPPS training on was deemed beneficial by some participants. The Social Worker shared, “*I think that some of the strategies just to take it at face value can be really overwhelming. Like, ‘Oh, how are we going to implement these?’ And I think we had an already in-house team that [the strategies] applied really well to, like, ‘Oh, we can focus on this team to use this stuff.’ I think that can be a really helpful way to do it. I think it would have been a little bit overwhelming to figure out how to have—like if we didn't have our team already existing, who to focus on*.”

In addition to having a specific unit or team to focus on, it is important to consider logistical aspects of infrastructure that are compatible with the TeamSTEPPS strategies. For example, School Psychologist 2 stated, “*We're all housed in the same area, so communication is easy. So, I think logistics is something that we would have to kind of think about, you know, proximity to each other. I mean, it is super easy for us to huddle because we're like two or three feet away from each other, whereas in some departments, you know, you're all the way down the hall or, you know, there's just physical barriers to communication, so that's something to consider, too.”* In some cases, it may be possible for teams to leverage aspects of their existing team organization to enable successful implementation; however, some teams will need to be planful in considering whether logistical aspects of the intervention are compatible with their existing team structure, as this could impact the success of the strategies.

#### 3.1.3 Individual characteristics

In the present study, two roles were identified within the “Individual” domain, which include the SMH Team Staff role and the role of Administrator. Unique characteristics of each of these roles were determined to impact implementation efforts.

##### 3.1.3.1 SMH team staff characteristic barriers

###### 3.1.3.1.1 Unmotivated innovation recipients

A common barrier identified by participants was that some SMH team members are less amenable to change. This poses a challenge to implementation efforts, particularly as it relates to strategies that alter typical workflow or responsibilities. The District Administrator illustrated this point, sharing, “*We had some other people that bought into it, and I think we had some other staff members that are instantly negative and resistant to change, so that's been a little bit harder to deal with.”*

The Team Leader shared that more established, veteran personnel are sometimes uniquely challenging to engage in these types of initiatives because of their preference for previous workflows, sharing, “*I think identifying and trying to like, formalize the leadership component, where, like, huddles are occurring has been sort of difficult… mostly because there's very senior counselors who have sort of been in the group they work in, they've been there for a while and they sort of have a good flow, you know. So, I think, you know, they feel like what they're doing works for them.”*

##### 3.1.3.2 SMH team staff characteristic facilitators

###### 3.1.3.2.1 Capable and motivated implementation team members

Several participants attributed the success of TeamSTEPPS implementation to a select team member or team members who were particularly motivated and committed to the TeamSTEPPS strategies. This commitment was illustrated by their willingness to speak up in favor and support of the strategies, remind the team of their commitment to this effort, and see implementation through.

The Principal illustrated the importance of having a champion of TeamSTEPPS on their SMH team, stating, “*And I know that [Team Member] is willing to do the time, even though he's not getting paid for that, right? So that's the tricky part, and in addition to everything else that he's doing. But he is hard set and definitely determined to get it… more efficient and have it be reflective across all meetings that we do and have it be kind of the model for how we do things.”*

Sometimes multiple champions of the strategies will arise on a single team, as the Assistant Principal shared, “*I do think having that meeting has pushed particularly those two team members to help us to get us to start laying out what our roles are. So, we did end up, like—we just recently had a training for Tier 2 intervention, and they were like, ‘No, we need more role clarity.’ So, our usual brush over of, ‘Oh, we can all help and pitch in,’ and—they were very adamant about making sure that we knew what each person's role is, and I think that had something to do with our TeamSTEPPS training.”*

###### 3.1.3.2.2 Capable and motivated implementation leads

Participants also recognized the role that SMH team leaders play in the successful implementation of initiatives like TeamSTEPPS. Team leaders facilitate implementation in various ways. For example, in addition to having a champion of TeamSTEPPS on their team, the Principal explained how they leverage their authority to support and empower the champion, stating, “*Even if I’m not available, he's moving forward with it, and I’m [going to] support him 100% in that […] I hope that they feel the same way, but that's—there shouldn't be any barriers.”*

Additionally, the Social Worker explained how they prioritize the integration of the TeamSTEPPS strategies into the structure of their team meetings, sharing, “*I definitely think it was driven kind of by—I run the meeting that we applied [the strategies] to, so I think that helped a lot. Like, there was just that connection that way.*”

##### 3.1.3.3 Administrator characteristic barriers

###### 3.1.3.3.1 Unmotivated leaders

Several participants raised concerns about the fact that their administration was not motivated to engage with this initiative. As such, despite SMH team efforts to implement the TeamSTEPPS strategies, they faced several challenges when interfacing with administration.

The District Administrator stated this issue clearly, “*I think if the leader doesn't buy into it, it doesn't work effectively. And we saw that, where one school, the principal was on board, and another was more reluctant so. It definitely does affect the outcome.”*

This point was re-iterated by the Assistant Principal, who shared, “*I feel like there isn't necessarily a buy-in. If there was a buy-in, there’d be action. So, it feels like a lot of lip service. You know, we do—we're using TeamSTEPPS. We want to organize and use these strategies, but when it comes down to it, they're not being utilized. It's the same thing over and over again. So, it really does feel like it—like we're just agreeing to agree and not really doing what we're saying that we're going to do.”*

##### 3.1.3.4 Administrator characteristic facilitators

###### 3.1.3.4.1 Motivated leaders

While a lack of leadership buy-in was a barrier in some schools, participants emphasized that *had* leadership been “bought in” to the TeamSTEPPS strategies, implementation would likely have improved. Indeed, in some settings, the administration *was* motivated, which resulted in successful implementation. The Team Leader shared, “*From the communication that I've had with the psychologists that worked with the [school] and the principal there, I know that … that the administrator is more involved, is helpful there and that they're more invested in [TeamSTEPPS], and I think I've seen from them an increased willingness to be able to utilize [the strategies].”*

The Assistant Principal emphasized how important this demonstrated support from leadership is, stating, “*Yeah, and I think that involvement is key because the words are always there. It always, ‘Sounds good,’ or they have new words to say, ‘Oh, this is how we organize our meetings,’ and, ‘This is what we're doing,’ but the actions aren't following what's being said. So, absolutely just making sure that things are actually done the way that we know would be useful and helpful would be wonderful*.”

One salient way that school administration can demonstrate support of initiatives like TeamSTEPPS is through modeling use of the strategies. As School Psychologist 1 shared, “*I mean, [it would be] nice in general if leadership [would] just refer to the strategies by name so that you know they are kind of supporting the use of the strategies in that way, instead of me abstractly talking about the ways we're going to run our teams better […] Having that language and I think having it reiterated from leadership again, it's a different power than me being someone equal to my colleagues. So having that push from leadership would be, I think, more helpful for implementation or buy-in from the rest of the team.”*

#### 3.1.4 The innovation

##### 3.1.4.1 Facilitators

###### 3.1.4.1.1 Innovation design

A key feature of the TeamSTEPPS intervention is the terminology used to label and describe the strategies that SMH teams may choose to adopt. Participants found that putting a name to these practices was inherently helpful and facilitated a shared language amongst team members that previously did not exist. This shared language allowed participants to recognize when they were engaged in evidence-based teaming practices, which reinforced use of the strategies.

Guidance Counselor 1 illustrated how this shared language could eventually lead to the natural sustainment of these strategies, stating, “*But when you call it something and you're like,” “Oh, we're huddling,” it's like you're really present. And so, I think it gives that opportunity to be present, then to really recognize like, “Oh, this is a strategy that I’m employing…*”

###### 3.1.4.1.2 Innovation relative advantage

Another facilitator of the TeamSTEPPS intervention was the effectiveness of the training at bringing awareness and attention to the needs of the team. Through discussion of effective teamwork strategies, teams were able to identify their shortcomings, which facilitated improvements moving forward.

The Assistant Principal emphasized this point, sharing, “*I do think there was an impact just in showing us to recognize that there is a need. Because you can't really fix anything that you don't really know is a need. But even though [having organized meetings with a clearer purpose] hasn't happened, I feel like just more members of our team working together to lay out the clarity and saying that we need to have meetings where things are clear [in terms of] why we're there and the goal of the meeting […] I really just do think that the organization of everything, the fact that we've recognized that we need more organization of what we're doing is going to be much [more] helpful in the future. It will be helpful in the future.”*

#### 3.1.5 Implementation process

##### 3.1.5.1 Barriers

###### 3.1.5.1.1 Planning

Time is a highly valued resource in school settings. Thus, concerns related to the scheduling of training and ongoing meetings to support TeamSTEPPS implementation proved to be a challenge. As the Principal shared, “*The key thing for us is having time to work and put those systems and processes in place. So, that's the hard part for us kind of moving forward. When do we actually sit together to do that?”*

##### 3.1.5.2 Facilitators

###### 3.1.5.2.1 Tailoring strategies

While the TeamSTEPPS model is comprised of a general training, it also includes opportunities for each team to identify areas of growth as well as the strategies that are most useful to the team. Participants emphasized the importance of this approach and recommended further approaches for tailoring the training content to the team. These recommendations included conducting observations of the SMH team prior to the training to better understand their typical team function. The Principal shared their experience, stating, “*[The research team member] sat in on [Student Service Team] meetings with us […] so that they could get a feel of what it currently looks like, so that they could provide, you know, feedback on how to make it better.*”

###### 3.1.5.2.2 Reflecting and evaluating

Participants also indicated that opportunities for reflecting and problem solving were critical to the successful implementation of TeamSTEPPS. Whether during the initial training, through formal or informal forms of communication, or at team meetings, these opportunities were viewed as important to ongoing implementation efforts.

The Team Leader emphasized the importance of regular touchpoints and opportunities for feedback to sustain implementation, sharing, “*So, having regular trainings outside of that, I think would be helpful sort of to review what's working, what's not working, and give feedback to each other and to the system itself.*”

Guidance Counselor 2 attended an initial individual TeamSTEPPS training for their SMH team before attending a larger, district-wide training. They emphasized how the review of TeamSTEPPS content and opportunity to hear and learn from colleagues at other schools was beneficial, stating, “*It was nice after we had the initial training being with the other centers and getting an idea of where they were in their thoughts. So, we would come together in our smaller group when we were in that semi-larger group, but it was still nice to hear what other people were thinking, too. So, I would say that was probably, of the trainings, that was probably the one that I got the most out of but that was also because we were already trained a little bit, and then we got to go a little bit deeper, and then with other people who were also trained.*” It is clear that to ensure successful implementation and future sustainment, regular check-ins about strategy use and problem solving are crucial.

### 3.2 Implementation research login model

**Figure 2:**
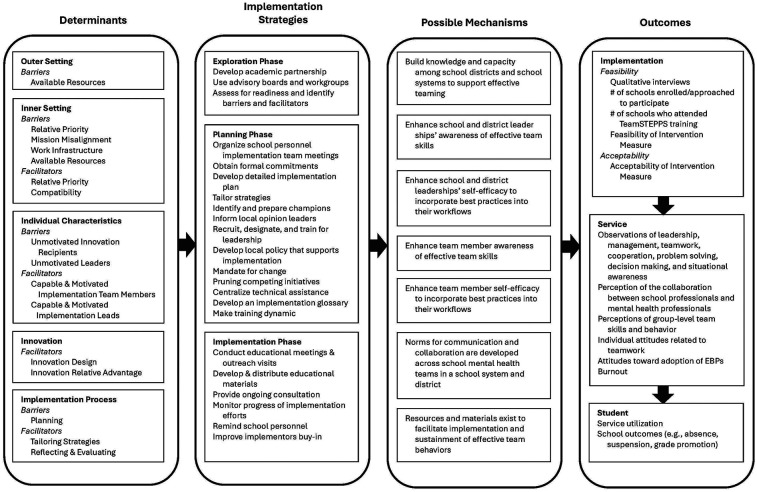
Implementation logic model.

Once the implementation determinants were identified and organized in accordance with the CFIR, the IRLM was developed to specify how these factors align with implementation strategies, mechanisms, and outcomes. Figure 2 depicts the final IRLM.

#### 3.2.1 Implementation strategies & associated mechanisms

Several implementation strategies that could be enacted to influence the identified determinants were selected from the SISTER (39). The hypothesized mechanism through which most proposed strategies would work is by building the knowledge and capacity of school leadership and SMH team members to implement effective teaming skills. This could be accomplished by providing dynamic training for leadership and SMH team members on evidence-based teaming practices, centralizing technical assistance for SMH team members implementing the TeamSTEPPS intervention, development and distribution of educational materials related to the intervention, and ongoing consultation about implementation supports or when teaming challenges arose. These proposed strategies and mechanisms are hypothesized to address barriers related to infrastructure, availability of resources, and lack of motivation. Furthermore, developing local policies that support implementation, establishing mandates for change, pruning competing SMH initiatives, and providing reminders of the teaming strategies to school personnel are proposed strategies aimed at targeting norms for communication and collaboration within school systems. These proposed strategies and mechanisms are hypothesized to address barriers related to the lack of prioritization of the initiative, mission misalignment, and lack of motivation.

#### 3.2.2 Outcomes

In the IRLM, outcome domains were identified and organized according to the IOF (41). These outcome domains are depicted sequentially with unidirectional arrows from “Implementation” to “Service” to “Student”-level outcomes. This illustrates that implementation outcomes often precede service and client related outcomes, setting the necessary preconditions for change. Proposed implementation outcomes included the Acceptability of Intervention Measure [AIM (42)], as well as several metrics of feasibility [i.e., number of schools recruited, enrolled, and that matriculated through the TeamSTEPPS training; the Feasibility of Intervention Measure, [(42); qualitative feedback]. Proposed service outcomes were related to effectiveness of the TeamSTEPPS intervention (e.g., observation of use of TeamSTEPPS strategies, perceptions of teaming, attitudes toward teamwork), as well as factors that may impact effective use of the TeamSTEPPS strategies (e.g., burnout). “Client outcomes,” which are phrased as student outcomes in the present study, would include proxies for satisfaction (i.e., service use) and “symptomatology”-related outcomes (i.e., absences, suspensions, grade promotion).

## 4 Discussion

This study was one of the first studies to examine perspectives of SMH team members, teachers, and administrators on factors that impacted implementation of a team science intervention within SMH teams. Using a systematic qualitative analytic process, barriers and facilitators to implementation were identified across the CFIR domains (30). As a result of this summative evaluation an IRLM was constructed to illustrate the conceptual links between participant identified implementation determinants, implementation strategies, mechanisms, and outcomes. The proposed IRLM provides a visual model documenting contextualized implementation factors of team science interventions for SMH teams.

Participants identified several barriers to the successful implementation of the adapted TeamSTEPPS intervention, including a lack of financing and resources (outer setting; inner setting), the intervention not being a relative priority, mission misalignment, poor work infrastructure to support implementation (inner setting), unmotivated innovation recipients and leaders (individual characteristics), and insufficient planning (implementation process). Facilitators that supported TeamSTEPPS delivery included positive local attitudes towards interdisciplinary teamwork (outer setting), the intervention being a relative priority for some team members, the compatibility of the intervention (inner setting), capable and motivated team members and leaders (individual characteristics), the design and relative advantage of the intervention (innovation), and tailored implementation strategies, along with reflection, and evaluation of the intervention (implementation process). While a limited but growing body of literature demonstrates the potential for team science interventions to improve SMH team dynamics (20–23), studies have yet to examine implementation determinants in accordance with CFIR. The barriers and facilitators identified in this study align with previously established factors that affect implementation of evidence based SMH services (43). Unsurprisingly, the same implementation challenges facing SMH service delivery are reflected in the dynamics of the SMH teams themselves. Indeed, these implementation determinants likely reflect the broader school system rather than any individual intervention. However, previous literature has emphasized that addressing the challenges of interorganizational collaboration is a critical prerequisite for effective and feasible SMH service delivery (43). Delivery of the adapted TeamSTEPPS intervention is one such solution to improving SMH services. Thus, identifying and testing strategies that leverage facilitators and overcome barriers to the delivery of TeamSTEPPS is a critical future direction, as this may enhance the effectiveness of SMH teams.

While IRLMs have guided program delivery and sustainment for various health-related concerns (44–48) including youth mental health (49), this study is the first to characterize a team science intervention for SMH teams using an IRLM. The field of implementation science has long recognized the need for greater transparency and openness to enhance the reproducibility of implementation science research (50). This is often challenging due to the complex interplay of theories and frameworks that guide implementation. The IRLM serves as an organizational framework to ground conceptual elements of implementation and to facilitate future implementation efforts and replicability (38). In this study, the IRLM specified several implementation determinants in accordance with CFIR. Implementation strategies were selected from the SISTER (39) and organized according to the EPIS framework (40). The identified strategies are intended to positively impact various outcome domains as specified by the IOF (41), including implementation (i.e., acceptability, feasibility), service (i.e., observation of use of TeamSTEPPS strategies, perceptions of teaming, attitudes toward teamwork, burnout), and student outcomes (i.e., service use, absences, suspensions, grade promotion). This comprehensive IRLM was informed by TeamSTEPPS recipients, offering critical insight into contextual factors that impacted implementation efforts. Consensus discussions and reference to an established literature base regarding implementation strategies (39), organizing frameworks (40), and outcomes (41) clarified the implementation process for this adapted team science intervention. Future examinations of the relationship between these factors will inform more effective implementation efforts and progress the field of implementation science.

The strengths of this study are important to contextualize alongside the limitations. Our findings may not generalize to other team science interventions or settings outside SMH teams. Furthermore, while participants informed the identification of implementation determinants, the remainder of the IRLM development involved the research team. Additionally, only one teacher was successfully recruited for participation in this study thereby further limiting the input of this informant group. Future research that incorporates the perspectives of community partners in the development of the IRLM will ensure accuracy, as community members often have critical expertise related to the implementation context. Additionally, information that could assist in further characterization of the sample was not gathered (e.g., class sizes of the participating schools, participant salaries, school procedures regarding confidentiality of student records). This may limit the generalizability of findings to other school contexts. Finally, this study's central contribution is the contextualized Implementation Research Logic Model (IRLM), which serves as an organizational framework to facilitate future implementation efforts and replicability; however, this IRLM was not statistically evaluated. Future work must center on validating and refining this model. Hybrid effectiveness-implementation trials should employ longitudinal and mixed-methods designs to quantify the causal pathways. Specifically, research should evaluate whether the proposed implementation strategies lead to the proposed mechanisms of change and whether these result in meaningful impacts on student outcomes. This would provide empirical validation for the sequential impact of team science interventions within SMH.

## Data Availability

The data is available upon reasonable request from the corresponding author.

## Footnotes

1 Henceforth, the terms “school mental health team” (SMH team) will be used to describe a team of school staff who are responsible for identifying and supporting students with behavioral and/or mental health concerns through direct service delivery and by connecting them to appropriate interventions and supports (Office of Planning, Evaluation and Policy Development, 2017).
