# Supplementary material for: Adapting TeamSTEPPS for school mental health teams: development of an implementation research logic model
Source: Front Health Serv. 2025 Dec 18;5:1675020. doi: 10.3389/frhs.2025.1675020 (PMC12756162; doi:10.3389/frhs.2025.1675020)

**Appendix A. Interview Guide**

Thank you for taking time to speak with me.

Today we want to hear about your experiences with TeamSTEPPS. We will be talking to several school personnel including student services staff, mental health providers, administrators, and educators to get a sense of their experiences with TeamSTEPPS, including whether they were satisfied with the strategies and whether they see it as something that could be beneficial in other schools. Hearing your thoughts and experiences is important to us because of the important role you play in your team’s provision of student support services and your experience this year with implementing TeamSTEPPS strategies.

Your participation in this interview is voluntary and you are under no obligation to discuss things that you do not feel comfortable sharing. All the information you provide will be kept confidential by our research team. I will be audio recording our conversation so that other members of our research team can listen to your comments later. We will then transcribe your comments and de-identify them. This is so that they cannot be traced back to you if used in future research products, like research papers. Once your comments are transcribed, we will destroy the recording.

Do you have any questions before we begin?

**Q1. When you think about the team that you work most closely with, who do you consider to be the members of your team?**

*Probes:*

- Who are the leaders that impact team function? (e.g., school administrators like principals or special education directors.)

**Q2. Describe your typical role and responsibilities.**

**Q3. Now I’d like to talk about TeamSTEPPS, the team training you participated in this year. To start, tell me about your experiences with the initial TeamSTEPPS training.**

*Probes:*

- What content from the training resonated with you or your team?
- How would you describe the compatibility of the initial training with your teams’ needs?
  - In what ways did it meet your expectations?
  - In what ways was it not useful?
- Was the time commitment reasonable?
- Was the initial training sufficient, or would you have preferred ongoing training to keep the program on track with the TeamSTEPPS approach?

**Q4. After the training, your team may have identified some key teamwork areas to focus on. Describe for me the TeamSTEPPS strategy(s) that your team chose to focus on and the process of implementation.**

*Probes:*

- How was it implementing that strategy(s)? Easy? Difficult?
  - What challenges did you face in implementing this strategy(s), if any?
  - What helped you to implement the strategy(s)?
- Describe for me the impact of the TeamSTEPPS strategy(s) on the challenges that your team was facing prior to implementation.
  - What did it help with, if anything?
  - What were the unanticipated effects?
- How have your team meetings changed, if at all, as a result of TeamSTEPPS?
- Describe any changes in yourself, your team members, and/or your teams’ dynamics as a result of TeamSTEPPS.
- Did you feel that you had the support you needed to implement the strategy(s)?
  - If so, describe for me what that support looked like.
  - If not, what types of support would have helped with implementation?
- How feasible has it been for you to meet the expectations of participating in the TeamSTEPPS program?

**Q5. Describe your satisfaction with the TeamSTEPPS strategy(s).**

*Probes:*

- Did you find that this strategy(s) was compatible with your team’s culture?
- With your admin’s culture?

**Q6. How likely are you to continue using this strategy(s) in the future?**

*Probes:*

- **If they do not plan to continue use,**
  - What influenced your decision to discontinue use of this strategy?
- **If they plan to continue use,**
  - What might get in the way of your continued use of the strategy?
  - What might help facilitate continued use of the strategy?

**Q7. Describe for me the role of leadership in the implementation and sustainment of TeamSTEPPS within your school.**

*Probes:*

- How have leaders facilitated your use of the TeamSTEPPS strategy(s), if applicable?
- How have leaders impeded use of the TeamSTEPPS strategy(s), if applicable?
- What do you need from leadership to sustain the TeamSTEPPS strategy(s)?

**Q.8 What suggestions do you have for other teams / schools thinking about using TeamSTEPPS?**

**Q9. Next, I’d like to review your responses on the survey measures you previously completed about your team and TeamSTEPPS. This is because surveys don’t always capture nuanced information.**

- **Do you think the strengths and challenges you told me about today were reasonably reflected in the surveys you completed?**
  - **If not**, what do you wish you would have indicated differently?

**Q10. Is there anything else you would like our team to know about your experience with TeamSTEPPS?**

This concludes our interview. Thank you for your time and participation.


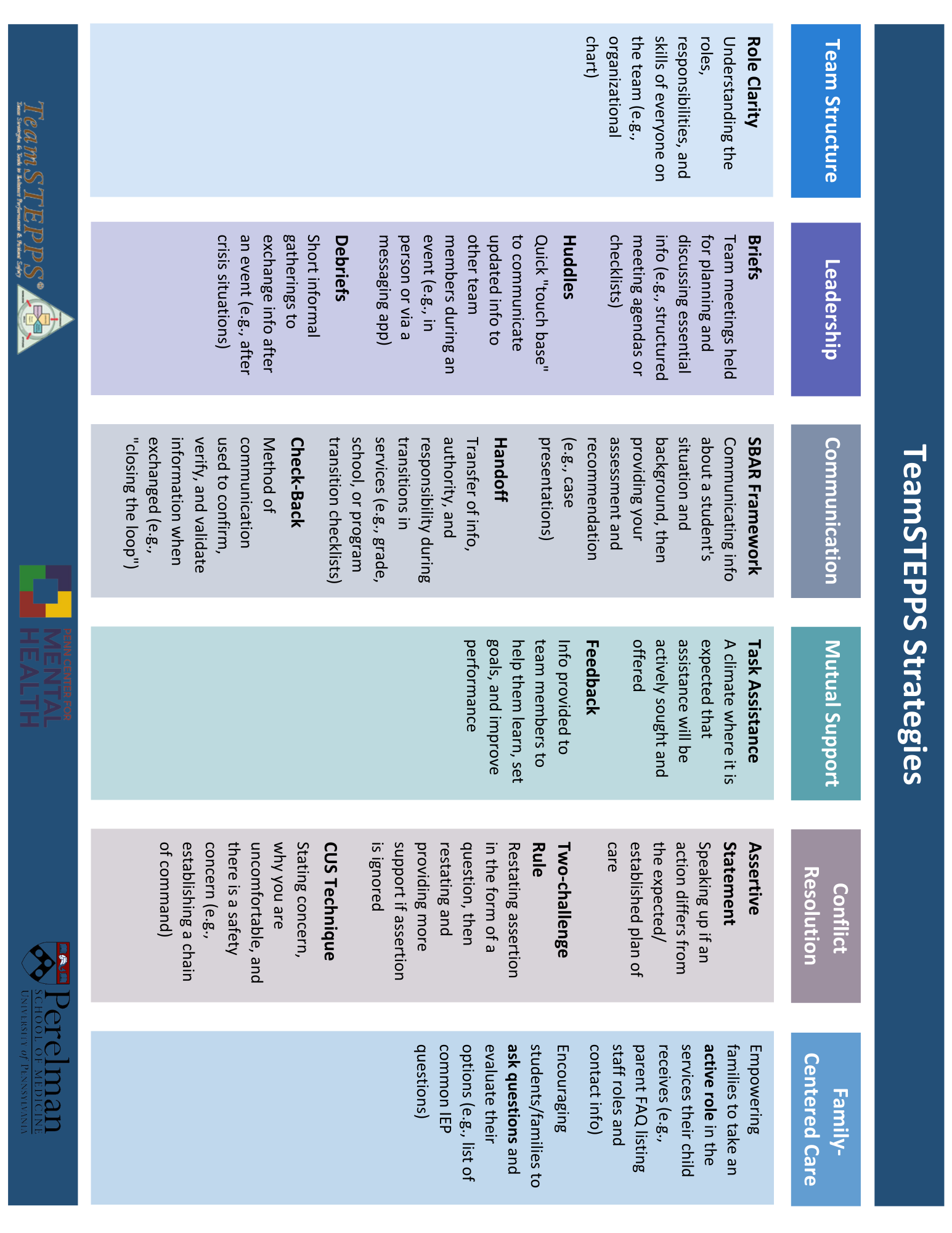

Supplement: Supplementary file 1 [file Supplementaryfile1.docx]
